# Environmental taxes, technological innovation and firm performance: Evidence from China's manufacturing firms

**DOI:** 10.1016/j.heliyon.2024.e31386

**Published:** 2024-05-15

**Authors:** Aiwu Zhao, Huizheng Zhang, Yilin Liu, Hongjun Guan

**Affiliations:** a School of Management Science and Engineering, Shandong University of Finance and Economic, Jinan, Shandong, 250014, China; b Institute of Marine Economics and Management, Shandong University of Finance and Economic, Jinan, Shandong, 250014, China; c Institute of Marine Economic Planning and Information Research, Hainan Academy of Marine and Fishery Sciences, Haikou, Hainan, 571126, China

**Keywords:** Environmental taxes, Technology innovation, Firm performance, Porter hypothesis, PSM-DID

## Abstract

Based on panel data from 2011 to 2019 for heavily polluting listed firms in the manufacturing industry, this paper examines the impact of environmental taxes on technological innovation and firm performance using the propensity score matching (PSM) and differences-in-differences (DID) methods. The empirical results show the following: (i) Firm performance and innovation quantity are positively affected by environmental taxes. The average effects of environmental taxes on firm performance and innovation quantity are 1.28 and 0.219, respectively. However, environmental taxes have no significant impact on innovation quality. (ii) A mechanism analysis reveals that innovation quantity plays a significant partial mediating role in the positive effect of environmental taxes on firm performance. (iii) Heterogeneity analysis shows that different environmental tax rates lead to a variation in innovation quantity and firm performance across regions. The positive effect of environmental taxes on innovation quantity is only confirmed in high-tax and low-tax areas. Meanwhile, high environmental taxes are related to better firm performance. Based on the research, policy recommendations are put forward to optimise environmental taxes, such as improving the environmental tax system and coordinating environmental tax and innovation policies.

## 1 Introduction

China's manufacturing industry accounted for approximately one-third of the GDP in 2021. The development of manufacturing has made significant contributions to China's industrialization and modernization process, but the extensive growth model of high pollution and high energy consumption has also exacerbated environmental pollution. Heavy polluting manufacturing enterprises account for 4/5 of the total industrial wastewater discharge. The COD emissions from the papermaking and food industries account for two-thirds of the total COD emissions, while the heavy metal emissions from the non-ferrous metallurgy industry account for nearly one-third of the total heavy metal emissions. Green transformation has become the key to sustainable development of the manufacturing industry.

In response to the increasingly severe environmental pollution, China is continuously strengthening its environmental regulations (ERs). On January 1, 2018, the Environmental Protection Tax Law of the People's Republic of China was officially implemented. Compared to traditional ERs, environmental taxes offer greater flexibility and stronger enforcement. Each region sets different rates for different pollutants and their own development accordingly. It is more flexible and explicit in terms of the target population, levy criteria and tax benefits, making the collection of environmental taxes more scientific and reasonable and easier to implement [1]. In addition, although the environmental tax policy is a market-oriented regulatory instrument based on price, it is also characterized by command-and-control regulations. The enactment of the Environmental Protection Tax Law has transformed China's environmental management from an administrative to a legal system, and therefore, taxation is more authoritative than sewage charges [2]. The compulsory nature of environmental taxes will make it more costly for firms to break the law, thus enabling it to be highly binding. Environmental taxes help reduce pollutant emissions [3]and can improve technological innovation in high-income and middle-income countries [4]. There has been a concern about whether environmental taxes are working as desired in China, which has great practical significance for policy implementation.

According to "Porter hypothesis", the cost pressures from ERs motivate firms to actively adjust their production patterns, which provides an incentive for technological innovation [5]. This would eventually improve the performance of the firm. Therefore, environmental taxes are entrusted with the important mission of promoting the manufacturing industry to achieve a win-win situation of economy and environment. However, neoclassical economics argues that the market competitiveness of enterprises will be undermined by the operating costs associated with environmental regulations [6]. At the same time, the increase in operating costs caused by environmental taxes may squeeze out the innovation resources of enterprises, leading to a decrease in innovation investment and having a negative impact on green transformation. Most of the existing research on the impact of environmental regulations on firm performance focuses on the overall environmental regulations, with less empirical research on the 2018 new version of environmental tax. Due to the dual attributes of mandatory and market-oriented environmental taxes, empirical research on their specificity can help reveal the underlying mechanisms by which ERs affect firm behavior. How environmental taxes affect firm performance in China's manufacturing industry, especially in heavily polluting industries? Can environmental taxes force enterprises to innovate and achieve green transformation? Can China's manufacturing industry under environmental tax constraints achieve a win-win situation under Porter's hypothesis? The resolution of these issues is of great significance for the high-quality development of China's manufacturing industry under the “dual carbon” constraints.

The structure of this paper is as follows: Section 1 presents the introduction; Section 2 provides the literature review; and Section 3 provides the theoretical analysis and hypothesis. Section 4 presents the research design; Section 5 presents the empirical results and analysis; Section 6 presents the robustness test; and the final section contains conclusions and suggestions.

## 2 Literature review

Traditional wisdom holds that ERs inevitably lead to higher operating costs and negative impacts on firm growth [7]. With the strengthening of ERs, heavily polluting firms face an even more severe external situation. Neoclassical economics argues that the market competitiveness of firms will be undermined by the operating costs associated with ERs. Some studies confirm that more stringent ERs lower the financial performance of heavily polluting firms [8,9]. China's export margins have also suffered as a result, and ERs have exerted a greater constraint on the exports of heavily polluting firms than on clean firms [10]. In addition, ERs act as a significant disincentive to total factor productivity in polluting industries [11]. The quality of products in heavily polluting industries has also declined [12]. Coping with the negative impact of increasingly stringent regulations would be a challenge for heavily polluting firms.

The "Porter hypothesis" breaks with conventional wisdom by suggesting that ERs could facilitate firm growth by promoting technological innovation [13]. ERs lead to the internalisation of environmental costs, which will motivate firms to proactively improve their innovation capabilities and ultimately gain a competitive advantage over others [14]. Numerous empirical results demonstrate the positive effects of ERs on firms' technological innovation. Song et al. (2019) [15]analysed provincial data from China, concluding that ERs played a facilitating role in technological innovation, which was characterised by spatial heterogeneity. Further research revealed a U-shaped relationship between ERs and technological innovation, with the impact on technological innovation evolving in an "incentive-disincentive-incentive" pattern as regulation is strengthened [[16], [17], [18]]. In addition, ERs also contribute to technological innovation through mediating factors. For example, Zhu et al. (2023a) [19]found that the compliance of ERs can accumulate external investment resources and improve innovation investment capacity for enterprises.

Several studies have found a correlation between higher firm performance and more advanced technological innovation [20]. Facing pressures from the environment and resources, green innovation can be used to improve the competitiveness of firms and provide them with new opportunities to develop in a green and intelligent direction [21]. Production process innovation and product innovation, as two aspects of technological innovation, can both act as intermediary factors to improve firm performance [22].Technological innovation not only promotes the financial performance of firms [[23], [24], [25]] but also effectively improves green total factor productivity [26]. Technological innovation is an indispensable link to a firm's sustainability [27]. However, Nie et al. (2023) [28]found that innovation at a low level of technology can have a negative impact on social welfare.

Further research found that the effect of ERs on technological innovation has uncertainty. Wang et al. (2022) [29]studied listed firms in China and concluded that both command-and-control and market-based ERs can positively affect firms' green technology innovation. However, not all ERs play a positive role. The empirical analysis of firms in China demonstrated that voluntary ERs contribute to technological innovation but that mandatory regulations have no obvious effect [30]. Regional heterogeneity in ERs showed that technological innovation in the eastern and central regions was not enhanced by command-and-control regulations but rather was limited [31]. Zhu et al. (2023b) [32]found that the incentive effect of tax incentives on ESG performance of enterprises is more significant in state-owned enterprises, enterprises in the eastern region, larger enterprises, enterprises with more concentrated equity, and enterprises with better internal control quality. Furthermore, Nie and Yang (2023) [33]pointed out that the larger the enterprise, the more active innovation investment becomes. Therefore, it remains to be further explored the exact effect of environmental taxes on specific firms in China.

Existing studies explained the uncertainty of the impact of environmental regulations on firm performance from both environmental costs and innovation drivers. Numerous scholars have studied the relationship between environmental regulation, technological innovation, and corporate performance based on the Porter hypothesis. These studies provide a theoretical basis for the study of this research. However, seldom research is focused on the impact of environmental tax. Due to the complex interaction between different policies, it is difficult to separately measure the net effect of environmental taxes in reality. The main contribution of this article is:

(i) Taking the implementation of environmental tax as a quasi natural experiment, PSM-DID method is employed to compare the changes of the experimental group and the control group after the implementation of environmental tax. The research reveals the net effect and mechanism of environmental tax on technological innovation and firm performance. What is more, by the comparison of the effects of different tax rates, the theoretical basis of the relationship between environmental regulation and innovation is further enriched. It can provide theoretical reference for improving the environmental policy system.
(ii) In response to the dilemma of economic growth and environmental protection faced by heavily polluting manufacturing enterprises in China, this study adopts listed companies with strong sensitivity to environmental regulations as empirical samples. The results can provide empirical reference for the green transformation of enterprises.

## 3 Theories and hypotheses

### 3.1 The effect of environmental taxes on technological innovation in firms

As an effective, preventive and long-term market regulation tool, environmental taxes exert more severe legal restrictions on firms' production plans. Technological innovation may be profoundly affected.

According to the "Porter hypothesis", innovation performance is sought to offset the costs of environmental management under appropriate ERs. "Innovation compensation" serves as an incentive for firms to innovate. The implementation of environmental taxes has brought about an increase in the unit cost of pollutant emissions, making it more expensive for heavily polluting firms to produce and undertake pollution control. Technological innovation can effectively reduce pollution and improve resource efficiency. Second, environmental taxes affect the direction of innovation in heavily polluting firms. In past production activities, the losses and hazards arising from the inadequate use of resources and pollutant emissions were not deeply understood by heavily polluting firms. Accordingly, they lacked experience in the control and treatment of pollutant emissions. With increased production costs and tighter controls on the environment, firms have to focus more on potential opportunities for environmental innovation. Meanwhile, the legal effect of environmental taxes embodies China's commitment to environmental pollution and the future direction of policies. Heavily polluting firms realise that green innovation can bring long-term economic benefits if integrated into development strategies. Finally, with the implementation of environmental taxes, firms have been forced to update their operating philosophy. In the past, more resource input was seen as an effective way to improve competitiveness, but it is now difficult for firms to make a living from such excessive development. While environmental tax policies have raised pollutant emission standards, the state has also formulated corresponding measures such as R&D subsidies and preferential taxation policies to encourage and support the technological innovation of firms, which helps to boost their incentive to innovate.

Faced with increasingly stringent environmental management, pollution control is the unshrinkable responsibility of heavy polluters. The two common means of pollution management are source control and end-of-pipe treatment [34]. Heavy polluters can not only reduce their pollutant emissions by improving their production processes but also treat the pollutants that have been produced to comply with emission standards. However, end-of-pipe treatment often fails to address the root of pollution problems, as it often only makes possible the transfer of pollutants and does not make better use of resources. In the long term, technological innovation from source control is more conducive to the sustainable development of heavy polluters. Source management places higher expectations on firms' technological innovation. The realisation of "green and intelligent manufacturing" requires firms to constantly upgrade their technology and improve the quality of innovation. Therefore, when discussing how environmental taxes affect technological innovation, the quantity of innovation is only part of the picture, and more emphasis should be placed on innovation quality.

In summary, environmental taxes provide sufficient motivation and a favourable social environment for the technological innovation of firms, which contribute to innovation quantity and innovation quality. Accordingly, the following hypotheses are proposed:

H1a Environmental taxes significantly improve the quantity of innovation in firms.

H1b Environmental taxes significantly improve the quality of innovation in firms.

### 3.2 The impact mechanism of environmental taxes on firm performance

Environmental taxes can affect firms’ behaviour by influencing their performance. According to traditional thinking, the implementation of environmental taxes entailed increased operating costs and decreased profits for companies. However, the competition and market conditions faced by companies change as a result of environmental taxes, prompting companies to adjust accordingly to market needs, which, in turn, will have an impact on firm performance.

The strong "Porter hypothesis" suggests that under appropriate ERs, managers will actively engage in innovative activities, and the gains from innovation could make up for compliance costs, thus improving firm performance [35]. Heavily polluting enterprises in the manufacturing sector are not only the main source of environmental pollution, but also the main bearer of environmental taxes, due to their characteristics of "high energy consumption, high emissions and high pollution". With rising environmental costs and increasingly stringent environmental regulations, heavily polluting enterprises have a stronger incentive to engage in technological innovation.

In addition, following national policy guidance, heavily polluting enterprises can deliver products that are more in line with market demand through green innovation. In the process, intangible assets such as the reputation and popularity of the firm will also be accumulated. Firm reputation is an important factor that influences consumers' purchasing decisions. A good firm reputation can not only increase consumer goodwill towards the firm, but the rational use of reputation is also conducive to reducing the production cost of the firm, thus improving the firm performance. To sum up, it is thus clear that technological innovation can be of great help in improving firm performance. Accordingly, these hypotheses are proposed as follows:

Hypothesis 2a Environmental taxes significantly improve firm performance.

Hypothesis 2b Environmental taxes improve firm performance by facilitating innovation quantity.

Hypothesis 2c Environmental taxes improve firm performance by facilitating innovation quality.

### 3.3 The heterogeneous effect of environmental tax rates on technological innovation

Environmental taxes allow a firm's environmental costs to be internalised as part of its production costs. As environmental tax policies differ from place to place, the difference in rates has led to varying degrees of production cost increases for businesses; thus, compromising the innovation behaviour of firms. Different firms may react differently when faced with different tax rates, For areas with low tax rates, the increased environmental costs of firms are not high. Technological innovation, on the other hand, is a long-term process that requires continuous investment. In addition, in this process, there is no necessary connection between inputs and outputs. This means that technological innovation is somewhat risky and may incur sunk costs. All of these will have a hindering effect on technological innovation. Firms may be more inclined to reduce costs through learning and imitation and be less active in innovation; additionally, their innovation structures are altered to a lesser extent. High environmental tax rates are always associated with high environmental costs for the firm. From a sustainability perspective, firms that pay high taxes are in greater need of "innovation compensation". Stimulated by the environmental taxes, it can significantly raise the technological innovation awareness of enterprises in high-tax areas, promote their green product innovation and green production technology innovation. The capacity for innovation is constantly being built up. Firms are, therefore, more inclined to carry out innovation activities for production optimisation and focus more on improving their performance with high-quality innovations. However, for companies to grow in the short term, the challenges of lower profits and lack of capital put them under more pressure. At this point, it is a better choice for managers to carry out imitative innovation. Accordingly, these hypotheses are proposed as follows:

H3a The effect of environmental taxes on innovation quantity varies with the tax rate.

H3b The effect of environmental taxes on innovation quality varies with the tax rate.

## 4 Research design

### 4.1 Data sources

In the Guidelines for Environmental Information Disclosure of Listed Companies issued by the Ministry of Environmental Protection in 2010, it was mentioned that regular disclosure of environmental information is a social responsibility of listed heavily polluting firms. As mandatory disclosure content, the availability and reliability of environmental taxes are guaranteed, so the empirical results are more convincing.

The basic and financial information required for this paper is mainly from the CSMAR and WIND databases. This paper refers to the literature and uses information on patent applications to characterise the technological innovation of firms [36]. The relevant data are collected from the State Intellectual Property Office (SIPO).

To ensure the study more referent, the original data are processed as follows: (1) firms with incomplete research data are eliminated; (2) firms that were forced to delist or given a mandatory delisting risk warning for poor management during the observation period are excluded; (3) the upper and lower tails of all observable variables are dropped by 1 % to keep the empirical results from being affected by extreme values.

### 4.2 Variable definition

(1) Dependent variables. The dependent variables in the study are technological innovation and firm performance. Technological innovation is discussed in both quantity and quality terms. Referring to previous studies, the total number and quality of patent applications are used to capture innovation quantity and innovation quality [37,38]. Patents in China include invention patents, utility patents and design patents. The natural logarithm of the total number of firm patent applications in the current year plus one is used to represent the quantity of innovation. Due to the difficulty of invention patents, invention patents as a percentage of total firm patent applications in the current period are chosen to characterise the quality of innovation. Return on total assets (ROA, i.e., net income for the current period/total assets for the previous period) is used to measure firm performance.
(2) Independent variable. The independent variable is environmental tax policies. A dummy variable is used to distinguish between the treatment and control groups. If the firm implemented environmental tax policies after 2018, the value is 1, which is the treatment group. If the firm did not implement environmental tax policies between 2011 and 2019, the value is 0, which is the control group.
(3) Control variables. Control variables are a set of observable variables that vary over time and affect dependent variables. Referring to the studies of Chen et al. (2022) [39]and Zhao et al. (2022) [40], seven indicators are selected as control variables: (1) firm size (SIZE); (2) capital structure (LEV); (3) cash flow level (CFO); (4) firm growth (GROWTH); (5) historical performance (LROA); (6) market power (MARKET); and (7) capital intensity (DENSITY). The definition of the control variables is listed in Table 1.

**Table 1:** Definitions of control variables.

Name	Symbols	Definition
Firm size	SIZE_i,t_	The natural logarithm of total firm assets
Capital structure	LEV_i,t_	Total liabilities/total assets
Cash flow levels	CFO_i,t_	Net cash flow from operating activities/total assets
Firm growth	GROWTH_i,t_	(Current period operating revenue minus Prior period operating revenue)/Prior period operating revenue
Historical performance	LROA_i,t_	Net income of the previous period/Total assets of the previous period
Market power	MARKET_i,t_	The logarithm of (sales revenue/operating costs)
Capital intensity	DENSITY_i,t_	The logarithm of (total fixed assets/number of employees)

### 4.3 Methodology

The PSM-DID analysis consists of two steps: propensity score matching (PSM) and the differences-in-differences (DID) method.

#### 4.3.1 PSM method

The implementation of environmental tax is not a natural experiment, so the grouping of enterprises does not guarantee that all enterprises have the same initial basic factors. To a certain extent, this will affect the regression results of the sample. Due to the different circumstances of the enterprises themselves, their response to the environmental taxes will also vary. For example, well-run large firms are better equipped for technological innovation than poorly run small firms. It is difficult to discern the impact of environmental taxes by directly comparing these two types of firms. The PSM eliminates the bias caused by sample selection in the policy effects test and makes the interpretation of the double-difference model more reasonable.

In PSM, firms are split into treatment and control groups according to whether environmental taxes are applied. The treatment group is "firms that have not implemented environmental tax policies from 2011 to 2017 and have implemented environmental tax policies from 2018 to 2019"; and the control group is "firms that have not implemented environmental tax policies from 2011 to 2019". The treatment group was matched to the control group with firm size (SIZE), capital structure (LEV), cash flow level (CFO), firm growth (GROWTH), historical performance (LROA), market power (MARKET) and capital intensity (DENSITY) as covariates.

The PSM method is designed based on matching estimates. Specifically, finding a particular firm j in the control group, firm j is as similar as possible to firm i in the treatment group in terms of firm size (SIZE), capital structure (LEV), cash flow level (CFO), firm growth (GROWTH), historical performance (LROA), market power (MARKET), and capital intensity (DENSITY). Then, firm j is matched with firm i, that is, $x_{i}=x_{j}$. In terms of the individual characteristics of the firm, assuming that whether a firm implements environmental taxes is fully determined by the covariates, the probability of implementing environmental taxes is similar for firm i and firm j. The PSM method calculates the propensity score P based on the seven observable variables of the firm and then compares the P values of the control and treatment groups to match firms with similar P values.

#### 4.3.2 DID method

The DID model is to be differenced separately in both time and grouping, which can avoid the endogeneity of the model to a certain extent. This method, combined with the PSM, can effectively eliminate the influence of other factors.

After PSM, the new treatment group and control group are obtained. A new dummy variable treated is generated. The value of treated for the new control group was 0, and the value of treated for the new treatment group was 1. At the same time, the time dummy variable time is generated. A value of 1 for time means 2018 and later years; a value of 0 for time means before 2018.

Based on the above, the basic regression model based on the DID is set out in this paper as follows:

(1) $$inv_{-}q_{i,t}=\alpha_{0}+\alpha_{1}treated_{i,t}\times time_{i,t}+\alpha_{n}X_{i,t}+Firm_{i}+Year_{t}+\varepsilon_{i,t}$$

(2) $$inv_{-}p_{i,t}=\beta_{0}+\beta_{1}treated_{i,t}\times time_{i,t}+\beta_{n}X_{i,t}+Firm_{i}+Year_{t}+\varepsilon_{i,t}$$

(3) $$ROA_{i,t}=\gamma_{0}+\gamma_{1}treated_{i,t}\times time_{i,t}+\gamma_{n}X_{i,t}+Firm_{i}+Year_{t}+\varepsilon_{i,t}$$

where i represents the firm and t represents the year. $inv_{-}q_{i,t}$, $inv_{-}p_{i,t}$, and $ROA_{i,t}$ are dependent variables used to measure the innovation quantity, innovation quality and performance of firm i in period t, respectively. $X_{i,t}$ is a set of time-varying observable control variables. It includes firm size, capital structure, cash flow level, firm growth, historical performance, market power, and capital intensity. $Firm_{i}$ and $Year_{t}$ are used to control the individual and time effects of the firm, that is, to control the effect of the firm's own characteristics and the effect of unobservable factors. $\varepsilon_{i,t}$ is the error term.

From Eq. (1), it is easy to see that for the control group (treated=0), the difference in innovation quantity $\left(inv_{-}q_{i,t}\right)$ is only affected by the time effect before and after the implementation of environmental taxes. For firms in the treatment group (treated=1), differences in innovation quantity before and after the implementation of the environmental taxes are affected not only by the time effect but also by environmental tax policies. By comparing the two, the net effect of the policy on the innovation quantity would be obtained as $\alpha_{1}$ in Eq. (1). $\alpha_{1}$, which is the coefficient to be concerned with. $\alpha_{1}$ represents the average change in innovation quantity of firms that implemented environmental taxes compared to firms that never implemented environmental taxes. If $\alpha_{1}$ is positive, it indicates that the policy benefits the innovation quantity of firms. In contrast, if $\alpha_{1}$ is negative, it indicates that the policy inhibits the innovation quantity of firms. Similarly, $\beta_{1}$ and $\gamma_{1}$ in Eqs. (2), (3) represent the net effect of environmental taxes on the quality of technological innovation and firm performance, respectively. In addition, the environmental tax rates in different regions are classified into high, medium and low levels, and heterogeneity analysis is conducted to investigate their effects on technological innovation and firm performance.

## 5 Analysis of empirical test results

### 5.1 Descriptive statistics

The preliminary statistical results of the samples are shown in Table 2.

**Table 2:** Descriptive statistics of variables.

Variable	Number of samples	Mean	Standard deviation	Minimum value	Maximum value
Innovations quantity	3474	2.849	1.508	0	6.385
Innovation quality	3474	1.978	1.307	0	5.308
Firm Performance	3474	4.18	5.81	−18.39	21.09
Firm size	3474	22.259	1.152	20.172	25.629
Capital structure	3474	0.404	0.209	0.043	0.91
Cash flow levels	3474	0.055	0.063	−0.125	0.228
Firm growth	3474	0.138	0.291	−0.418	1.647
Historical performance	3474	4.45	5.682	−15.67	21.31
Market power	3474	0.394	0.394	−0.015	1.865
Capital intensity	3474	12.975	0.822	11.088	14.929

From Table 2, the average values for innovation quantity and quality in China are 2.849 and 1.978, respectively. Innovation quality is less than innovation quantity. Overall, there is still huge potential for innovation in China.

### 5.2 PSM results

In the study, the treatment group for PSM is 206 firms that started to implement environmental tax policies in 2018, and the control group is 180 firms that did not implement environmental tax policies from 2011 to 2019.

The propensity scores of the samples were estimated by the logit model, and the weights were determined by radius matching. After excluding the samples that did not satisfy the "common support assumption", 3457 samples remained. The matched PSM results are required to satisfy a balance test with as little difference as possible in observable variables between the treatment and control groups. The results of the balance test are shown in Table 3.

**Table 3:** Results of the covariate balance test.

Covariates	Unmatched U/Matched M	Average value		% Deviation	Deviation Reduction	T-test	
		Treatment group	Control group			t-value	P-value
SIZE	U	22.426	22.067	31.7	93.4	9.30	0.000
	M	22.426	22.403	2.1		0.61	0.544
LEV	U	3.8218	5.1688	−23.8	87.3	−7.02	0.000
	M	3.8218	3.6502	3.0		0.93	0.353
LROA	U	0.43795	0.36509	35.5	94.9	10.41	0.000
	M	0.43795	0.43423	1.8		0.54	0.588
CFO	U	0.0563	0.05392	3.8	90.7	1.11	0.269
	M	0.0563	0.05652	−0.3		−0.11	0.914
GROWTH	U	0.13154	0.14572	−4.9	77.0	−1.43	0.152
	M	0.13154	0.12828	1.1		0.33	0.738
MARKET	U	0.31091	0.48808	−45.6	96.4	−13.56	0.000
	M	0.31091	0.31721	−1.6		−0.60	0.549
DENSITY	U	13.153	12.771	47.7	94.6	14.03	0.000
	M	13.153	13.174	−2.6		−0.76	0.447

Compared to the prematching period, the differences between the treatment and control groups are significantly narrower in seven aspects after matching, and the absolute value of the standard deviation for each observable variable is less than 10. Therefore, the radius matching estimates can be considered reliable with the appropriate covariates and proper matching methods in this paper. At this point, the characteristics of the treatment and control groups are similar, and their probabilities of implementing environmental tax policies in 2018 are similar, so comparisons can be made.

### 5.3 DID results

#### 5.3.1 Parallel trend test

DID is one of the common methods used to evaluate the effects of policies. Its prerequisite is that the trends of the independent variables remain consistent between the control and treatment groups before the policy shock. Therefore, to verify the appropriateness of the DID model, this paper uses an event study approach to examine parallel trends in the dependent variables. First, the time dummy variable period is generated for the years 2011–2019, with observations taken as 1 in the current year and 0 in other years. Then, the interaction items between the time dummy variables period and the processing group dummy variable treated are generated and added to the DID model for regression. The coefficient of this interaction term represents the difference between the treatment and control groups in period t. The parallel trend test models for the three dependent variables are shown as Eqs. (4), (5), (6), respectively.

(4) $$inv_{-}q_{i,t}=\alpha_{0}+\sum_{t=2011}^{2019}\alpha_{t}treated_{i,t}\times period_{i,t}+\alpha_{n}X_{i,t}+Firm_{i}+Year_{t}+\varepsilon_{i,t}$$

(5) $$inv_{-}p_{i,t}=\beta_{0}+\sum_{t=2011}^{2019}\beta_{t}treated_{i,t}\times period_{i,t}+\beta_{n}X_{i,t}+Firm_{i}+Year_{t}+\varepsilon_{i,t}$$

(6) $$ROA_{i,t}=\gamma_{0}+\sum_{t=2011}^{2019}\gamma_{t}treated_{i,t}\times period_{i,t}+\gamma_{n}X_{i,t}+Firm_{i}+Year_{t}+\varepsilon_{i,t}$$

**Fig. 1:**
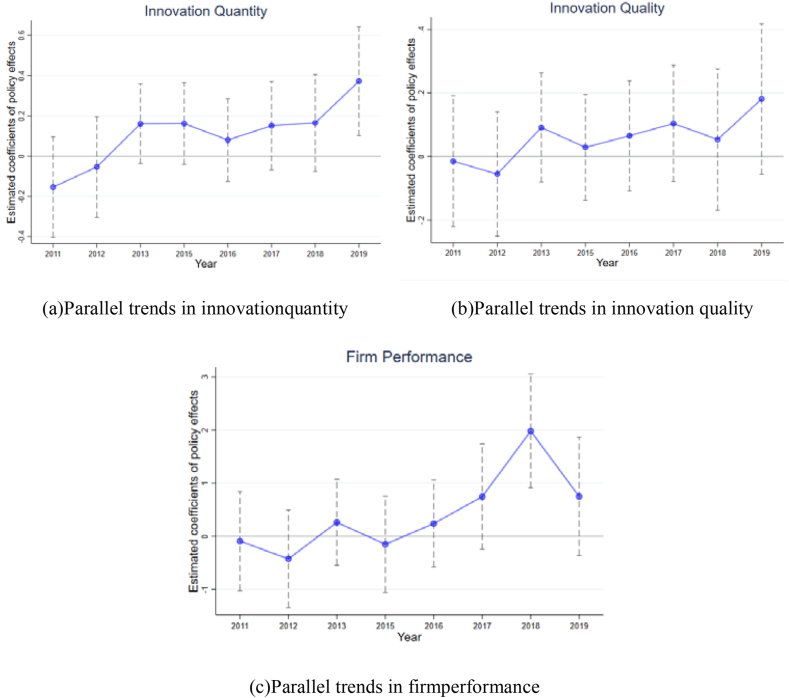
Parallel trend test results.

Taking 2014 as the benchmark, $\alpha_{t}$, $\beta_{t}$ and $\gamma_{t}$ represent a series of estimated values from 2011 to 2019. The other variables are the same as Eqs. (1), (2), (3). The estimated results of $\alpha_{t}$, $\beta_{t}$ and $\gamma_{t}$ at the 95 % confidence level are plotted in Fig. 1.

According to Eqs. (4), (5), (6), parallel trend tests were conducted for innovation quantity, innovation quality and firm performance, and the results are shown in Fig. 1.From Fig. 1, the differences in innovation quantity and firm performance between the treatment and control groups are insignificant before the implementation of environmental taxes, while there are significant differences in the trends between the two after the environmental taxes. Thus, the hypotheses of parallel trends regarding innovation quantity and firm performance hold, ensuring the validity of the DID results.

In addition, the difference in innovation quantity between the control and the treatment groups is not obvious in the current period of policy implementation, and there is a significant difference in the second year. This shows that environmental taxes have a lagging effect on innovation quantity. The reason for the lag may be that patent applications take a long time to be approved, so there is a lag in patent applications. For innovation quality, the trends between the two groups are largely consistent before the policy shock. However, after environmental taxes, there is still no significant difference in the growing trends of innovation quality, indicating that environmental taxes have little impact on innovation quality, and the hypothesis of the parallel trend is not valid.

According to the results, China's technological innovation is still mainly imitative. Although innovation quantity has improved, innovation quality is not good. It will take a long time to transition from imitative innovation to independent innovation. As imitative innovation is less difficult and less time-consuming, in the short run after implementation of environmental taxes, firms tend to improve their competitiveness through imitative innovation in the face of increased production costs. Therefore, in the year of policy implementation, firms in the treatment group performed significantly better than those in the control group. Over time, however, there is no significant difference between the control and treatment groups in terms of firm performance in the first period after policy implementation. This implies that high-quality innovation is key to gaining and maintaining long-term competitive advantages under a "carbon peaking and carbon neutrality" strategy. Imitative innovation has difficulty maintaining its competitive advantage.

#### 5.3.2 Basic model regression

Based on Eqs. (1), (2), (3), the basic regression results are presented in Table 4.

**Table 4:** Basic regression test.

	(1)	(2)	(3)	(4)	(5)	(6)
	ZL_ALL	ZL_ALL	ZL_1	ZL_1	ROA	ROA
$ROA_{i,t}=\sigma_{0}+\sigma_{1}treated_{i,t}\times time_{i,t}+\sigma_{2}inv_{-}q_{i,t}+\sigma_{n}X_{i,t}+Firm_{i}+Year_{t}+\varepsilon_{i,t}$	0.215**	0.219**	0.084	0.085	2.335***	1.280***
	(0.094)	(0.091)	(0.091)	(0.088)	(0.517)	(0.381)
EN_SIZE		0.523***		0.458***		0.191
		(0.076)		(0.073)		(0.341)
LORA		0.012***		0.012***		0.135***
		(0.004)		(0.004)		(0.035)
LEV		−0.063		0.039		−13.569***
		(0.189)		(0.160)		(1.473)
CFO		0.016		0.057		15.715***
		(0.322)		(0.278)		(1.821)
GROWTH		0.002		0.038		3.963***
		(0.060)		(0.050)		(0.367)
MARKET		−0.036		−0.092		6.483***
		(0.161)		(0.155)		(1.215)
DENSITY		−0.041		−0.097**		−0.127
		(0.056)		(0.047)		(0.288)
_cons	2.465***	−8.463***	1.536***	−7.297***	5.106***	3.333
	(0.047)	(1.728)	(0.041)	(1.622)	(0.240)	(7.554)
N	3457	3457	3457	3457	3457	3457

Note: *, **, *** denote coefficients are significant at the 10 %, 5 % and 1 % levels respectively.

According to the results of Columns (3) and (4) in Table 4, for innovation quality, the coefficients of the interaction terms are insignificant, while the hypothesis of a parallel trend on innovation quality does not hold, so the research on innovation quality has not been further explored. The reason may be that after the implementation of the environmental taxes, heavily polluting enterprises are forced to take the initiative to improve resource utilization efficiency, increase green productivity and reduce pollutant emissions. However, due to the financial and time constraints, heavily polluting firms may start with low-cost imitation innovations.

Models (1), (3), and (5) are the estimated results without considering other control variables, and Models (2), (4) and (6) are the estimated results with other control variables. From Models (1), (2), (5) and (6), the regression coefficients of the interaction terms are all significantly positive, regardless of the inclusion of other control variables. This shows that environmental taxes significantly improve innovation quantity and firm performance, which is consistent with Hypotheses 1a and 2a.

The analysis of Model (2) concludes that firm size and historical performance play a significant role in promoting the quantity of innovation. Factors such as cash flow level, firm growth, historical performance and market power all positively affect firm performance. In other words, higher net cash flow from day-to-day operations, better historical performance and more robust growth are more conducive to firm performance. However, the impact of capital structure on firm performance is negative, indicating that high debt is detrimental to firm performance.

The variance inflation factor (VIF) analysis is also conducted, and the results show that the VIF values of the variables are below 10, so there is no serious multicollinearity.

#### 5.3.3 The mediating effect test of technological innovation

The "Porter hypothesis" argues that environmental taxes can enhance the competitiveness of firms by promoting technological innovation, which leads to higher firm performance. On this basis, this paper takes the innovation quantity as a mediating variable and establishes the mediating effect model of environmental taxes and firm performance concerning the method of Wen et al. (2014) [41]. Through Eqs. (1), (3), (7), the mediating role of technological innovation is tested jointly. The results are listed in Table 5.

(7) $$ROA_{i,t}=\sigma_{0}+\sigma_{1}treated_{i,t}\times time_{i,t}+\sigma_{2}inv_{-}q_{i,t}+\sigma_{n}X_{i,t}+Firm_{i}+Year_{t}+\varepsilon_{i,t}$$

**Table 5:** Mediating effect test of technological innovation.

	(7)	(8)	(9)
	ROA	ZL_ALL	ROA
treated ×time	1.280***	0.219**	1.230***
	(0.381)	(0.091)	(0.381)
ZL_ALL			0.228**
			(0.090)
LROA	0.135***	0.012***	0.132***
	(0.035)	(0.004)	(0.035)
LEV	−13.569***	−0.063	−13.554***
	(1.473)	(0.189)	(1.467)
CFO	15.715***	0.016	15.711***
	(1.821)	(0.322)	(1.822)
GROWTH	3.963***	0.002	3.963***
	(0.367)	(0.060)	(0.365)
MARKET	6.483***	−0.036	6.491***
	(1.215)	(0.161)	(1.220)
DENSITY	−0.127	−0.041	−0.118
	(0.288)	(0.056)	(0.288)
_cons	3.333	−8.463***	5.259
	(7.554)	(1.728)	(7.523)
N	3457	3457	3457

Note: *, **, *** denote coefficients are significant at the 10 %, 5 % and 1 % levels respectively.

The results of Models (7), (8) and (9) in Table 5 show that innovation quantity exerts a significant positive mediating effect between environmental taxes and firm performance. The effect value of innovation quantity is 0.050 (0.219 * 0.228), accounting for 3.90 % of the total effect of 1.280. Hypothesis 2b is verified. It can be seen that environmental taxes force enterprises to engage in technological innovation to reduce pollutant emissions and hence improve firm performance. The innovation driven effect of Porter's hypothesis exists in heavily polluting enterprises in China.

#### 5.3.4 A test of heterogeneity in the impact of environmental taxes on innovation quantity

Environmental taxes are not subject to unified standards nationwide. Instead, based on the set minimum, the tax administration authority is delegated to the local government. Each provincial administrative region is entitled to set its own levy criteria according to local economic development, resource and environmental restrictions. This move makes environmental tax policies more suited for different regions. This paper classifies each region into high-, medium- and low-tax areas based on the criteria for environmental taxes, as shown in Table 6.

**Table 6:** Environmental tax rates by region.

Tax level	Region	Description of the levy criteria
High	Beijing, Hebei, Henan, Jiangsu, Shandong, Shanghai, Sichuan, Tianjin	Atmospheric pollutants: more than $2 per pollution equivalent, up to $12. Water pollutants: more than $2 per pollution equivalent, up to $14.
Medium	Guangdong, Guangxi, Hainan, Guizhou, Heilongjiang, Shanxi, Hubei, Hunan, Zhejiang, Chongqing	Atmospheric pollutants: $1.40 or more per pollution equivalent, up to $3.50. Water pollutants: more than $1.40 per pollution equivalent, up to $3.
Low	Yunnan, Fujian, Anhui, Gansu, Jilin, Jiangxi, Liaoning, Ningxia, Qinghai, Shaanxi, Xinjiang	Atmospheric pollutants: more than $1.20 per pollution equivalent, up to $2.80. Water pollutants: from $1.40 per pollutant equivalent, up to $3.50.

This paper refers to the heterogeneity study by Hope et al. (2020) [42]. The sample of the treatment group is divided into three groups of high, medium and low tax rates and regressed against the control group. The DID regression results are shown in Table 7, where Models (10), (11) and (12) are the results for high-, medium- and low-tax areas, respectively.

**Table 7:** Testing the effects of different environmental tax rates on technological innovation.

	(10)	(11)	(12)
	ZL_ALL	ZL_ALL	ZL_ALL
treated ×time	0.254**	0.068	0.379**
	(0.111)	(0.126)	(0.149)
_cons	−9.286***	−8.864***	−8.676***
	(2.101)	(1.985)	(2.174)
N	2368	2179	2062

Note: *, **, *** denote coefficients are significant at the 10 %, 5 % and 1 % levels respectively.

As shown in Table 7, the impact of tax rates on innovation quantity varies across regions, so Hypothesis 3a is verified. From Model (10) and Model (12), the coefficients are significantly positive at the 5 % level, implying that environmental taxes significantly boost innovation quantity in both high- and low-tax regions. According to Model (11), in medium-tax rate regions, environmental tax policies have a nonsignificant effect on innovation quantity. For high-tax areas, higher environmental costs "force" firms to increase profits through technological innovation. The cost of producing in low-tax areas is not significantly different from what it was in the past, following the "fee to tax". The cost of environmental protection does not overwhelm the production resources of firms. However, the shift to a greener economy in China has become inevitable. For the long-term development of firms, managers are willing to invest more in technological innovation, resulting in an increase in innovation quantity. For medium-tax areas, the increase in environmental costs is lower than that of high-tax rate regions. The internal and external pressures faced by firms are not sufficient to motivate innovation. At the same time, the innovative behaviour of the firm may also be constrained by increasing environmental costs. As a result, there is no obvious influence on the innovative behaviour of the firm.

#### 5.3.5 A test of heterogeneity in the mediating effect of innovation quantity

Similar to the above heterogeneity study, this paper also explores the effects of different tax rates on both firm performance and the mediating role of innovation quantity. The results of the DID regressions are shown in Table 8, where Models (13) and (14) are for high tax rate areas, Models (15) and (16) are for medium tax rate areas, and Models (17) and (18) are for low tax rate areas.

**Table 8:** Testing the effects of different environmental tax rates on firm performance.

	(13)	(14)	(15)	(16)	(17)	(18)
	ROA	ROA	ROA	ROA	ROA	ROA
treated ×time	1.671***	1.606***	0.957*	0.935*	0.816	0.764
	(0.429)	(0.432)	(0.519)	(0.508)	(0.587)	(0.593)
ZL_ALL		0.258**		0.325***		0.137
		(0.120)		(0.120)		(0.121)
_cons	3.120	5.520	−0.975	1.904	−2.501	−1.313
	(8.959)	(8.958)	(8.500)	(8.453)	(9.184)	(9.183)
N	2368	2368	2179	2179	2062	2062

Note: *, **, *** denote coefficients are significant at the 10 %, 5 % and 1 % levels respectively.

Model (13) in Table 8 shows that environmental taxes increased firm performance by 1.671 at the 1 % level of significance in high-rate areas. In Model (15), the environmental taxes increased the performance of medium-rate firms by 0.957 at the 10 % level of significance. In Model (17), the environmental taxes had no significant effect on firm performance in the low-rate areas. Considered together with the results of innovation quantity, it is in high tax areas that innovation quantity exerts a significant positive mediating effect. It follows that a higher intensity of environmental taxes can induce firms to realise the "Porter hypothesis" effect by improving the quantity of innovation.

## 6 Robustness tests

To verify that the results are not accidental, the robustness test is conducted from two aspects: replacing the matching method and random grouping.

### 6.1 Replacement matching method

The estimation results in Table 4 are based on the radius-matching method. In this paper, one to three nearest-neighbour matching and kernel matching methods are proposed to test the robustness of the model. Models (19) and (20) are the regression results obtained by one-to-three nearest-neighbour matching. Models (21) and (22) are the regression results obtained by the kernel-matching method. The results are presented in Table 9.

**Table 9:** Basic regression results for different matching methods.

	(19)	(20)	(21)	(22)
	ZL_ALL	ROA	ZL_ALL	ROA
treated ×time	0.221**	1.068***	0.222**	1.280***
	(0.097)	(0.410)	(0.091)	(0.381)
EN_SIZE	0.557***	0.047	0.533***	0.191
	(0.076)	(0.344)	(0.078)	(0.341)
LROA	0.012***	0.135***	0.012***	0.135***
	(0.004)	(0.033)	(0.004)	(0.035)
LEV	−0.163	−12.973***	−0.063	−13.569***
	(0.201)	(1.474)	(0.190)	(1.473)
CFO	−0.179	15.556***	0.026	15.715***
	(0.332)	(1.939)	(0.323)	(1.821)
GROWTH	0.040	3.996***	0.003	3.963***
	(0.064)	(0.403)	(0.060)	(0.367)
MARKET	−0.048	6.710***	−0.036	6.483***
	(0.168)	(1.341)	(0.161)	(1.215)
DENSITY	−0.036	−0.144	−0.042	−0.127
	(0.058)	(0.323)	(0.056)	(0.288)
_cons	−9.215***	6.310	−8.648***	3.333
	(1.689)	(7.979)	(1.751)	(7.554)
N	2872	2872	3457	3457

Note: *, **, *** denote coefficients are significant at the 10 %, 5 % and 1 % levels respectively.

Table 9 shows that the coefficients of the interaction terms are significantly positive regardless of the matching method used. The estimation results on innovation quantity are all significantly positive at the 5 % level, and those on firm performance are all significantly positive at the 1 % level. Therefore, the reliability and stability of the baseline regression results can be demonstrated.

### 6.2 Placebo test

**Fig. 2:**
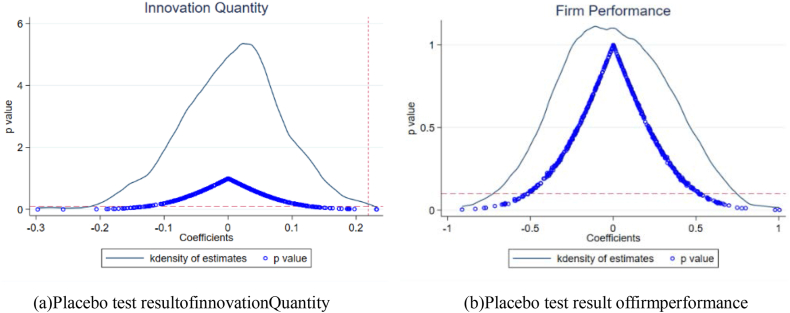
Placebo test results.

With reference to Cai et al. (2016) [43], this paper further demonstrates the robustness and credibility of the results by randomly generating control and treatment groups to exclude the effects of some uncontrollable factors on technological innovation and firm performance. In the sampling process, 206 firms are randomly selected from the samples as the treatment group for implementing environmental tax policies, while the others are the control group. 500 random samplings are conducted. After each random sampling, the interaction term is reconstructed to estimate the effect of policy implementation. When the estimation results are insignificant, the impact of other uncontrollable factors is small, and the results of this paper are credible. The coefficient distributions of the interaction terms and their P values are plotted. According to the results in Fig. 2, these 500 estimations are close to zero, and most of them are not significant, indicating that the results are unlikely to be influenced by other factors.

## 7 Conclusions and policy recommendations

Environmental taxes put heavily polluting firms in China's manufacturing industry under even tougher control. How to break through the dual constraints of the environment and core technology is the key to achieving green transformation and upgrading. This paper applies econometric theories and methods to explore the effect of the "Porter hypothesis" in China. Heavily polluting firms in China's manufacturing industry are selected as the research object. Using the PSM-DID method based on quasinatural experiments, an empirical study was conducted to explore whether environmental tax policies promote technological innovation by firms, with the aim of reducing pollution and achieving economic growth. The National Innovation-Driven Development Strategy (NIDDS) projects that China will reach the goal of becoming an innovation power by 2050, which suggests that attention should be given not only to innovation quantity but also to innovation quality. Therefore, the study of technological innovation of firms is carried out in two aspects: innovation quantity and innovation quality.

The results show that environmental taxes are an effective driver of technological innovation and firm performance (see Table 4, Table 5). This is consistent with the conclusion of Zhu et al. (2020) [44]that the carbon tax mechanism promotes green innovation in enterprises and promotes their green development. However, the positive effect of environmental taxes on firm technological innovation is more on innovation quantity, while there is no significant contribution to innovation quality (see Fig. 1). Second, environmental taxes improve firm performance by facilitating technological innovation. This proves the applicability of the "Porter hypothesis" in China. In particular, appropriately higher environmental taxes could better leverage innovation to improve firm performance. Third, environmental taxes affect innovation quantity and firm performance through the tax rate (see Table 7, Table 8). Regions with high and low taxes better enjoy the incentives of environmental taxes for technological innovation, while the medium tax rate has not played a notable role. Meanwhile, high environmental taxes are related to better firm performance.

Based on the analysis of the above results, the policy recommendations proposed are as follows:

(1) Develop appropriate standards for environmental taxes and deepen the reform of the environmental taxes. As shown in Table 7, Table 8, technological innovation and firm performance by region are associated with environmental tax rates. Environmental tax policies with appropriate standards can effectively incentivise firms to innovate and are important for the health and sustainability of China's economy. In the phase of economic transformation and industrial restructuring, the government of China should develop environmental tax policies that correspond to local economic development and environmental pollution according to its characteristics to maximise the benefits in terms of "environment" and "economy".
(2) Encourage independent innovation and promote innovation quality. Table 4 shows that environmental taxes have stimulated the growth of innovation quantity but do not significantly improve innovation quality by firms. To be a "manufacturing power", China must conquer core technologies in major fields and place a high priority on innovation quality. The government should vigorously advocate for independent innovation and enhance firm awareness of the importance of protecting innovation achievements. At the same time, the review of innovation achievements should be strengthened, and the relevant legal system should be improved. A good environment for the protection of intellectual property rights is conducive to the formation of a correct sense of competition and inspires firms to innovate on their own.
(3) Provide support for technological innovation and improve environmental tax policies. According to Table 7, environmental tax policies have no significant effect on innovation quantity in medium-rate regions. As the environmental costs of tax policies will squeeze the research funds of firms and discourage their technological innovation, financial support for technological innovation should be enhanced in policy development. To reduce the risk of carrying out innovation activities, the R&D subsidies of firms could be increased. More policy support, such as tax exemptions and preferential treatment, could be provided to reduce the difficulty of financing firms' innovative activities. In addition, companies need to attract more highly qualified researchers. By improving the talent pool for technological innovation and establishing a set of effective incentive mechanisms, the research potential of talent can be more effectively tapped.

## Ethical approval

Not applicable.

## Consent to participate

Consent to participate of this paper was obtained from all authors.

## Consent to publish

Consent to publish of this paper was obtained from all authors.

## Funding

This research was funded by “the Special Funds for 10.13039/501100010040Taishan Scholar Project”, China (Grant No. tstp20231229).

## Data availability statement

Data and materials are available from the authors upon request.

## CRediT authorship contribution statement

**Aiwu Zhao:** Supervision, Project administration. **Huizheng Zhang:** Writing – original draft, Resources, Formal analysis. **Yilin Liu:** Methodology, Formal analysis, Data curation. **Hongjun Guan:** Writing – review & editing, Funding acquisition.

## Declaration of competing interest

The authors declare that they have no known competing financial interests or personal relationships that could have appeared to influence the work reported in this paper.
